# Effect of Different Cleaning Regimes on Biofilm Formation of Acrylic-Based Removable Orthodontic Appliance: A Randomized Clinical Trial

**DOI:** 10.1155/2023/9920850

**Published:** 2023-10-12

**Authors:** Safa I. Khawwam, Dheaa H. Al-Groosh

**Affiliations:** Department of Orthodontics, College of Dentistry, University of Baghdad, Iraq

## Abstract

**Objectives:**

This study aimed to evaluate the effects of different cleaning regimes of acrylic-based removable orthodontic appliances on bacterial biofilm formation and whether the surface modification, i.e., polished acrylic fitting surface, reduces biofilm formation.

**Materials and Methods:**

This double-blind, parallel, randomized clinical trial involved thirty-nine orthodontic patients indicated for removable orthodontic appliances. The patients were allocated into three groups according to the cleaning method: brushing with a denture brush and chlorhexidine (CHX) toothpaste, Lacalut cleaning tablet, and a combination of both cleaning methods. Each patient wore an upper removable appliance containing eight wells fitted with eight detachable acrylic tiles (four polished and four unpolished) for seven days. Five types of oral microbiota were evaluated using selective growth media and biochemical tests. The biofilm cleaning efficacy was assessed using the colony-forming unit (CFU) and scanning electron microscopy (SEM). *Statistical Analysis*. Data from the CFU using different cleansing regimes were compared, following log transformation, using one-way analysis of variance (ANOVA). The polished and unpolished tiles were compared for biofilm formation on each cleansing method using an independent *t*-test.

**Results:**

There was no significant difference among the three cleaning methods on the polished or unpolished tiles. However, in polished tiles, streptococci were significantly reduced in all cleaning methods, whereas staphylococci and *Staphylococcus aureus* were markedly decreased in brushing and combination cleaning methods. However, the total number of anaerobic bacteria was significantly reduced in polished tiles using the combination method only.

**Conclusions:**

Polishing the fitting surface of an acrylic-based orthodontic appliance reduced the tested bacterial biofilm formation and may enhance cleaning efficiency. Brushing and combination methods showed superior cleaning effects compared to cleaning tablets. This trial is registered with NCT05707221.

## 1. Introduction

People seeking orthodontic treatment have increased not only to correct their malocclusions but also to improve mastication, speech, appearance, overall health, comfort, and self-esteem [1]. However, various adverse effects were encountered during orthodontic treatment with fixed or removable appliances [2]. It was found that polymethyl methacrylate (PMMA), a preferred material for acrylic-based appliances, was prone to microbial colonization and opportunistic biofilm adherence in the oral cavity [3] due to several factors such as material porosity and surface roughness, poor denture hygiene maintenance, and night-time wearing [4]. Removable orthodontic appliances increase the prevalence of *Candida* in the oral cavity and may induce *Candida* infection in compromised patients [5]. Staphylococci and transient microbiota were also recovered from orthodontic retainers and the oral cavity of the retainer's wearers. These opportunistic pathogens, which were associated with a wide range of infections, including abscesses in multiple organs, endocarditis, gastroenteritis, and toxic shock syndrome [6], became a part of the oral flora in an orthodontic patient [7, 8]. Furthermore, the prevalence of streptococci, one of the main causes of dental caries [9], was significantly increased in patients with orthodontic appliances [10].

Maintaining good oral hygiene is integral to successful orthodontic treatment [11, 12]. Previous studies have been conducted to investigate the effects of different cleaning protocols of removable orthodontic appliances on biofilm growth. These include chemical materials such as denture cleaners, enzymatic solutions, chlorhexidine, sodium hypochlorite, or homemade solutions containing vinegar or citric acid [13] and mechanical approaches, including brushing (with water, soap, toothpaste, or abrasives) and ultrasonic therapy. Although mechanical cleaning with brushes is affordable, it may not be indicated in patients with poor motor coordination and dexterity [14]. On the other hand, using cleaning solutions may facilitate the removal of adherent microorganisms present in inaccessible niches within the rough surface texture of acrylic-based appliances [15].

This clinical trial was designed to compare the effectiveness of different cleaning methods, including brushing with chlorhexidine (CHX) toothpaste, the use of cleaning tablets, and a combination of both protocols on biofilm removal on acrylic-based removable orthodontic appliances, and to assess whether polishing the fitting surface has an impact on biofilm adhesion and growth.

## 2. Methods

### 2.1. Trial Design

This double-blind, parallel, randomized clinical trial was approved by the Ethical and Board Committee of College of Dentistry, University of Baghdad (issue no. 598/April 2022), and registered under protocol ID ClinicalTrials.gov: NCT05707221 (date of registration: 31 January 2023) according to the CONSORT 2010 statement [16].

### 2.2. Participants

Thirty-nine subjects were recruited from the Department of Orthodontics at the College of Dentistry. Those included 24 females and 15 males aged 16–31 (a mean of 23.1 years). Those patients fulfilled the following criteria [17]:

- Patients who were clinically fit and healthy and had no history of systemic diseases
- Patient who had been scheduled to wear an upper removable orthodontic appliance due to minor tooth discrepancies
- The presence of full upper permanent dentition
- Patients who had no history of sensitivity to persulfate or chlorhexidine
- Caries-free patients with good oral hygiene

The exclusion criteria are as follows:

- Participants who were under steroid-based medications, broad-spectrum antibiotics, or antibacterial mouthwash two months before the experiment
- Patients who were smoking or tobacco eaters
- Mouth breather
- Pregnant or lactating female

The study protocol was explained to the participants verbally and in writing, and written informed consent was obtained from each participant.

### 2.3. Randomization

A computer random number generator developed a simple, nonstratified randomization of three groups (https://www.graphpad.com/quickcalcs/randomize2/). An independent person gave each number a study code to develop the allocation table, which included the study code and the allocation group. The table was kept sealed from the investigators until data measurement and analysis were completed.

### 2.4. Allocation Concealment

It was accomplished using an opaque and sealed pack marked with a treatment allocation code. Each pack contained the allocated cleaning materials and an instruction card that an independent person gave.

### 2.5. Interventions

#### 2.5.1. Construction of Removable Appliance with Acrylic Tiles of Different Surface Textures

**Figure 1:**
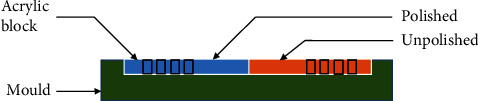
Acrylic tiles made from polished and unpolished acrylic sheets.

Acrylic tiles (5.5 mm × 1 mm) of two surface textures were made from an acrylic block of 1 mm thickness using a mould of silicone rubber (Rema® Sil silicone duplicating material, Dentaurum, Ispringen, Germany) [18]. Half of the sheet was polished using a conventional polishing technique until a glossy surface appeared, whereas the remaining was kept without modification. Tiles of 5.5 mm in diameter were bored using a trephine bur (Figure 1). The polished and unpolished tiles were colour-coated from their seated surfaces.

**Figure 2:**
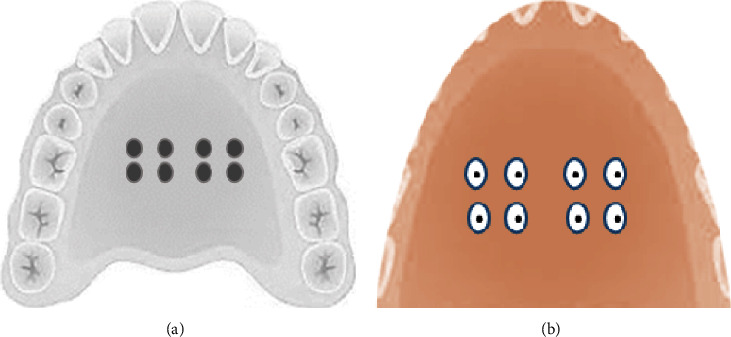
Construction of a modified acrylic-based orthodontic appliance. (a) Study model with metal discs; (b) fitting surface of the removable acrylic appliance with a chimney-like structure.

After obtaining the consent form, an impression was taken on the maxillary arch using alginate impression material (Hydrogum 5, Zhermack, Badia Polesine, Italy). The negative replica was then poured using type IV stone to make a study model and coated with a separating medium (Separating Agent, Shanghai New Century Dental Materials Co., Ltd., Shanghai, China). Eight detachable metal discs of 5.5 mm × 1.2 mm were distributed on the palatal side of the stone model (Figure 2(a)). Next, according to the manufacturer's instructions, an acrylic-based removable appliance was constructed using Orthocryl (Orthocryl, Dentaurum, Ispringen, Germany) with a powder-to-liquid ratio of 2.5 : 1. Before setting, a sprue or chimney-like structure was created through the acrylic dough to facilitate metal discs' removal (Figure 2(b)). The metal discs were removed through the chimney-like structure to create wells within the palatal fitting surface of the appliance.

**Figure 3:**
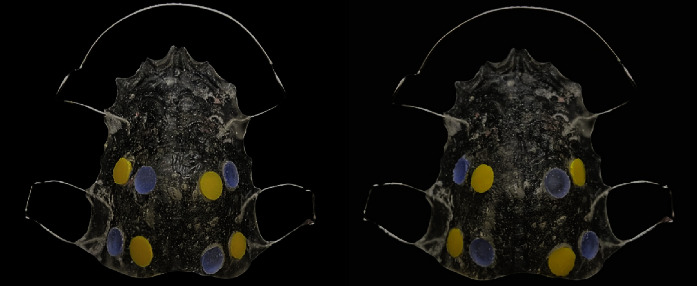
Each appliance contained eight wells to house: four (5.5 mm) diameter polished tiles (yellow) and four similar diameter unpolished tiles (purple). The allocation of the polished tiles to the left or right sides, anterior or posterior of the removable appliance, was decided by a randomized number sheet; the unpolished tiles were subsequently inserted into the wells on the contralateral side.

The acrylic tiles, with different roughnesses, were randomly seated and fixed into the wells using sticky wax (Figure 3). The wire components of the removable appliance were fabricated using a 0.7 mm diameter of stainless steel wire (spring hard, Dentaurum, Ispringen, Germany) according to the design required.

The appliance was sterilized using 115 V UV light in a UV sterilization hood (Dahian Labtech Co., Laminar hood, Korea) for 15 minutes [19] before being inserted into the patient's mouth.

#### 2.5.2. Patient Grouping

The patients were randomly divided into three groups:

1. Lacalut cleaning tablet group: Patients were instructed to use one tablet (LacalutDent, Lacalut, Germany) dissolved in 150 ml of tap water. The appliance was soaked for 20 minutes and then washed with tap water.
2. Brushing with CHX toothpaste group: Patients were instructed to clean the appliance with a denture brush (Foramen Denture Brush, Spain) loaded with CHX toothpaste (LacalutActive, Lacalut, Germany) for one minute and then washed with tap water.
3. Combination group: Patients were instructed to clean the appliance using a brush and CHX toothpaste, similar to the brushing group, followed by soaking it in the cleaning tablet solution for 20 minutes. The appliance was then washed with tap water.

The patients were asked to wear the appliance for seven days and follow the instructions and the sealed cleaning method assigned.

#### 2.5.3. Sample Collection

**Figure 4:**
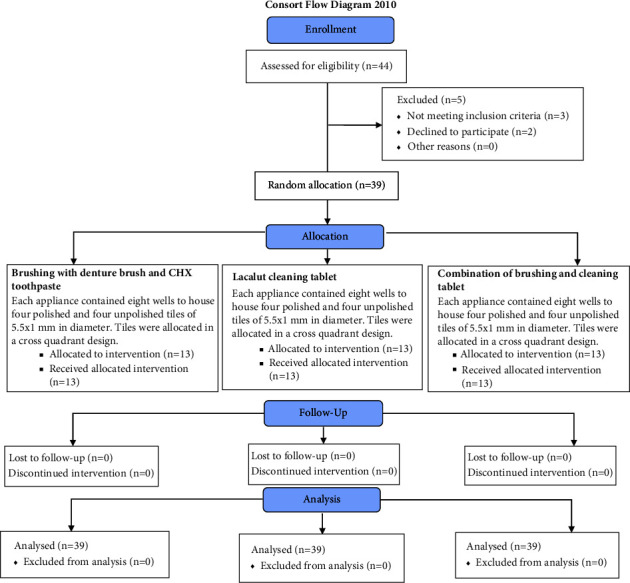
CONSORT flow diagram of subject randomization and selection criteria.

A CONSORT flow diagram illustrating subjects' flow during the clinical trial was followed (Figure 4).

After seven days of wearing the appliance, three acrylic tiles of each roughness category were gently removed from their wells using a metal pin through the chimney-like structure without disturbing the biofilm. The tiles were held using a sterile tweezer and immersed twice in a 15 ml bijou tube containing phosphate buffer saline (Sigma-Aldrich) to remove the planktonic bacteria and then inserted into a 1.5 ml bijou tube containing 1 ml PBS and vortex mixed for 1 minute to disseminate the biofilm and create a homogenous solution. A ten-fold serial dilution was carried out using PBS, and 50 *µ*L of the suspension was used for the plate culturing method [20]. The samples were cultured into three culture media: Mitis Salivarius agar (Liofilchem, Italy), a selective agar for streptococci, was incubated anaerobically for 48 hours at 37 C° in a candle jar [21]. Blood agar (Oxoid, England) was incubated anaerobically for 48 hours at 37 C° in the anaerobic jar with a gas bag to give the total anaerobic count. Mannitol salt agar (Oxoid) was incubated aerobically for 48 hours at 37 C° for staphylococci [22].

Bacteria were distinguished by colony morphology and were characterized by Gram reaction [23] and other confirmatory tests, including catalase and coagulase tests [24]. A specialist blinded to the study was responsible for the bacterial colony count. After statistical analysis, the allocation concealment table was revealed.

#### 2.5.4. Scanning Electron Microscope (SEM)

The fourth acrylic tile (of each roughness category) was removed from the appliance aseptically and immersed carefully in a Petri dish containing PBS (Sigma-Aldrich). The sample was prepared for SEM using a previously suggested protocol [18].

### 2.6. Statistical Analysis

Data from the colony-forming unit (CFU) using different cleansing methods were compared, following log transformation, using one-way analysis of variance (ANOVA). The polished and unpolished tiles were compared for biofilm formation on each cleansing method using independent *t*-tests. The significance level was set at a *p* value of ≤0.05 with a 95% confidence interval.

## 3. Results

The results showed no significant difference among different cleaning methods regarding staphylococci, streptococci, and total anaerobic bacterial biofilm on polished tiles (*p* value >0.05) (Table 1).

Similarly, there was no significant difference among different cleaning methods regarding the tested bacterial biofilm on unpolished tiles (Table 2).

**Figure 5:**
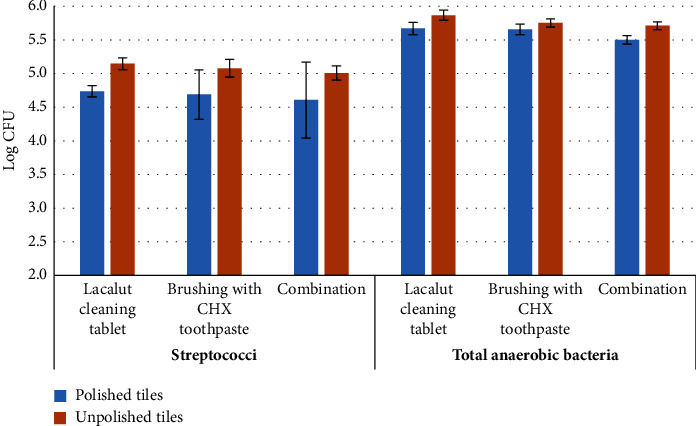
Bar chart of streptococci and total anaerobic bacteria among groups on polished and unpolished tiles. Error bars represent the standard error of measurements for 13 patients in three separate sample runs (*n* = 39).

**Figure 6:**
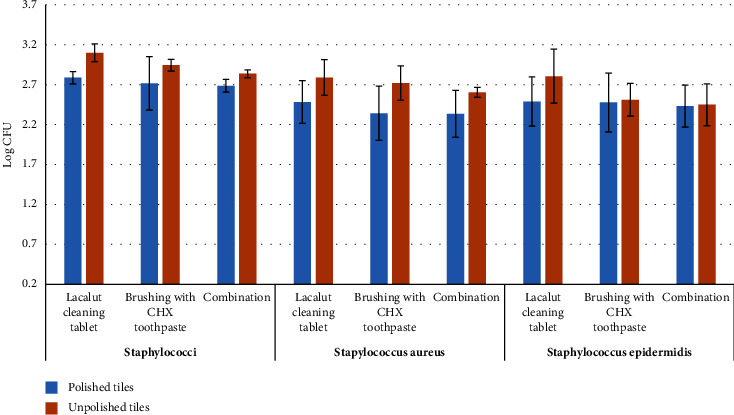
Bar chart of staphylococci among groups on polished and unpolished acrylic tiles. Error bars represent the standard error of measurements for 13 patients in three separate sample runs (*n* = 39).

Table 3 and Figures 5 and 6 show the effect of cleaning methods on bacterial biofilm between the polished and unpolished tiles. The polished tiles showed less biofilm than the unpolished tiles, regardless of the cleaning method. The effectiveness of brushing and the combination cleaning method was significantly superior in reducing the biofilm in polished tiles. This was true for staphylococci, *S. aureus*, and streptococci (*p* = 0.028, 0.022, and 0.025, respectively, for the brushing method; *p* = 0.035, 0.028, and 0.033, respectively, for the combination method); however, the total anaerobic bacterial counts were significantly reduced in the combination cleaning method only (*p* = 0.017). The Lacalut cleaning tablet reduced streptococci effectively in the polished tiles only (*p* = 0.003).

**Figure 7:**
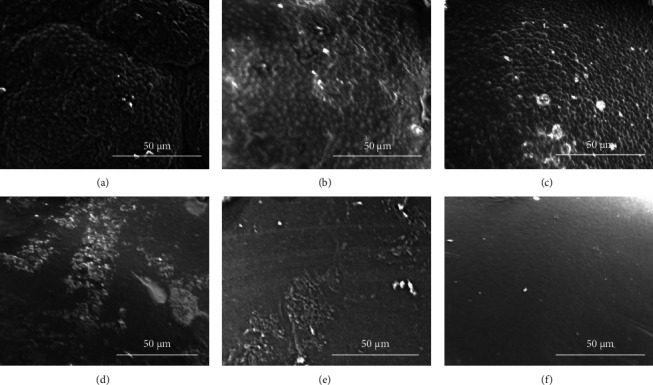
SEM micrographs of retrieved acrylic tiles. (a–c) The unpolished acrylic tiles cleaned with the Lacalut cleaning tablet, brushing with CHX toothpaste, and a combination of methods, respectively. (d–f) The polished acrylic tiles cleaned with the Lacalut cleaning tablet, brushing with CHX toothpaste, and a combination of methods, respectively, using conventional laboratory polishing procedures.

The SEM micrographs of the polished and unpolished tiles revealed that the unpolished acrylic tiles showed a similar amount of bacterial biofilm, irrespective of the cleaning method. However, all cleaning methods in polished tiles were effective and demonstrated fewer biofilm aggregates (Figure 7). Indeed, the surface substratum of the combination cleaning method on the polished tiles appeared clear (Figure 7(f)), followed by the brushing and the cleaning tablet methods (Figures 7(d) and 7(e), respectively).

## 4. Discussion

This randomized clinical trial was designed to compare the effects of mechanical and chemical cleansing methods on bacterial biofilm attached to acrylic-based removable orthodontic appliances. It was reported that the prevalence of opportunistic pathogens was higher in PMMA-based intraoral devices such as feeding appliances in cleft lip and palate patients, orthodontic retainers, and prosthodontic prostheses [7, 25, 26]. Previous studies compared the mechanical or chemical cleaning methods solely on biofilm-removing efficacy [27]; however, to the author's knowledge, no information was available on the effects of polishing the fitting surface of the acrylic-based orthodontic appliances on biofilm reduction using these cleaning methods apart from a laboratory-based attempt to reduce the amount of biofilm by modifying the surface texture of these appliances using an artificial mouth device, i.e., the constant depth film fermenter [18].

The sample size was estimated based on a pilot study of 8 participants' appliances for cultivated staphylococci counts, with a mean of 1200 CFU for the Lacalut cleaning tablet and 800 CFU for the brushing group. A total of 12 patients were needed per group to yield an alpha value of 0.05 with a study power of 80%. A 10% dropout was estimated, which renders the total sample of 39 patients. The sample size was comparable to many previously published randomized clinical trials conducted on biofilm formation on dentures and disinfection methods.

This study showed no significant difference among the cleaning methods on bacterial biofilm in unpolished groups. A similar result was found in the polished groups. It was found that the acrylic resin, i.e., the autopolymerized acrylic resin in this study, was apron to bacterial adhesion due to several surface properties, including surface hydrophobicity, surface free energy, and surface roughness [18]. The “built-in” surface irregularities of the PMMA increase the physical surface area and offer niches that protect bacteria from dislodging forces and promote bacterial adherence and colonization [28]. The results came in accordance with Albanna et al. [17], who found that chemical cleansing tablets (Retainer Britet, Kukist, and Coregat) following mechanical cleaning with brushing and water did not differ significantly in reducing the CFU of *S. mutans*, *S. epidermidis*, and *S. aureus* in Essix retainers. Furthermore, Kasibut et al. [29] found that there was no significant difference between cleaning acrylic retainers with a cleaning tablet (Polident Pro Guard and Retainer®) and brushing regarding the prevalence of *S. mitis*, the most common genus across all taxonomic categories, *S. gordonii*, *N. flavescens*, *S. sanguinis*, and *R. dentocariosa*. Moreover, Chang et al. [30] found that mechanical cleaning with brushing and CHX gel (Corsodyl dental gel), brushing with fluoride-containing toothpaste (Colgate), and immersion in mouthwash containing CHX (Corsodyl) exhibited no significant difference on *S. sanguis*, *Actinomyces naeslundii*, MRSA, and *Candida albicans*. Moreover, Oliveira Paranhos et al. [31] studied mechanical cleaning by brushing and dentifrices and chemical cleaning with alkaline peroxide solution for removable prostheses; they reported a similar efficacy in reducing the bacterial biofilm of *Staphylococcus aureus* and *S. mutans* on acrylic resin in an *in vitro* study.

However, previous clinical trials suggested that brushing reduced denture biofilm formation compared with immersion in a peroxide solution [32]. This disagreement could be due to the difference in the experimental setting, where the clinical study may have a different variety of microbial populations, in addition to patients' perceived instructions, i.e., verbal and visual instructions, which might impact the outcomes. Furthermore, the difference in patients' dexterity could explain the disparity in cleaning outcomes using the brushing method [33, 34].

Regarding the comparison of the polished and unpolished tiles, the data showed that the polished surface significantly facilitated *S. aureus* and streptococci removal in the brushing and combination cleaning method groups. At the same time, the cleaning tablet revealed its effectiveness against streptococci on polished tiles. Furthermore, the cleaning effect of the combination method was effective for the total number of anaerobic bacteria on the polished tiles. This result aligns with Farhadifard et al. [35], who found that the effectiveness of brushing and denture cleaning tablet methods in cleaning removable orthodontic appliances was higher than brushing alone. This could be justified by the surface free energy and the hydrophobicity of the PMMA, which played an important role in the initial bacterial attachment and successive biofilm formation [18, 34]; however, this may not enhance biofilm retention.

Furthermore, the Lacalut cleaning tablet contains potassium monopersulphate (MPS), a broad-spectrum disinfectant that oxidizes the bacterial protein capsids and evacuates cell content; its action depends on the contact time and concentration [35]. Lacalut Active toothpaste contains CHX digluconate, an effective cationic antimicrobial agent with broad antibacterial activity against Gram-positive and Gram-negative bacteria, fungi, and certain viruses [36]. It has been demonstrated that 2% chlorhexidine has antimicrobial activity against *S. aureus*, *E. coli*, and *Salmonella* [36]. CHX may synergize the cleaning tablet effects in the combination method.

The SEM micrographs showed the superior cleaning results of the combination method, followed by the brushing and the cleaning tablet methods; this was true for the polished and unpolished surfaces; however, there was a dispersed distribution of the bacterial aggregates in the polished samples, which showed a relatively cleaner surface. This agreed with the finding reported by Al Groosh et al. [18], who demonstrated that MRSA was detectable in microscopic surface irregularities of the unpolished samples and aggregated initially in the rough areas within the polished autopolymerized acrylic samples.

### 4.1. Study Limitations

There are limitations encountered in the current study. These include the microbial diversity between individuals and the response of bacterial species to individual cleaning methods.

Furthermore, this study was conducted on clinically fit and healthy patients, and further investigation may widen the scope of its novelty, i.e., patients with diabetes, immune suppressive medications, or those with obturators such as “baby feeding plates,” orthodontic retainers, prosthodontic prostheses, etc.

## 5. Conclusion

The study found that polishing the acrylic surface significantly reduced bacterial biofilm, regardless of the cleansing method used. Furthermore, brushing and the combination cleaning methods showed superior cleaning efficacy compared to the Lacalut cleaning tablet for all tested bacteria in polished acrylic samples.

## Figures and Tables

**Table 1:** Comparison of bacterial biofilm on polished tiles among the three cleaning methods using the ANOVA test.

Bacterial strain	Cleaning method	*N*	Mean	Standard error	*P* value
Staphylococci	Lacalut cleaning tablet	13	6.11 × 10^2^	1.29 × 10^2^	0.143
	Brushing with CHX toothpaste	13	5.19 × 10^2^	2.03 × 10^2^	
	Combination	13	4.84 × 10^2^	9.58 × 10^1^	

*Staphylococcus aureus*	Lacalut cleaning tablet	13	3.04 × 10^2^	7.34 × 10^1^	0.327
	Brushing with CHX toothpaste	13	2.19 × 10^2^	1.18 × 10^2^	
	Combination	13	2.15 × 10^2^	5.50 × 10^1^	

*Staphylococcus epidermidis*	Lacalut cleaning tablet	13	3.08 × 10^2^	1.32 × 10^2^	0.581
	Brushing with CHX toothpaste	13	3 × 10^2^	1.08 × 10^2^	
	Combination	13	2.69 × 10^2^	5.95 × 10^1^	

Streptococci	Lacalut cleaning tablet	13	5.46 × 10^4^	8.50 × 10^3^	0.377
	Brushing with CHX toothpaste	13	5.03 × 10^4^	1.13 × 10^4^	
	Combination	13	4.05 × 10^4^	1.12 × 10^4^	

Total anaerobic bacteria	Lacalut cleaning tablet	13	4.68 × 10^5^	7.79 × 10^4^	0.403
	Brushing with CHX toothpaste	13	4.55 × 10^5^	7.12 × 10^4^	
	Combination	13	3.17 × 10^5^	4.29 × 10^4^	

**Table 2:** Comparison of bacterial biofilm on unpolished tiles among the three cleaning methods using the ANOVA test.

Bacterial strain	Cleaning method	*N*	Mean	Standard error	*P* value
Staphylococci	Lacalut cleaning tablet	13	1.25 × 10^3^	4.05 × 10^2^	0.657
	Brushing with CHX toothpaste	13	8.76 × 10^2^	1.797 × 10^2^	
	Combination	13	6.88 × 10^2^	8.32 × 10^1^	

*Staphylococcus aureus*	Lacalut cleaning tablet	13	6.15 × 10^2^	1.55 × 10^2^	0.865
	Brushing with CHX toothpaste	13	5.23 × 10^2^	1.49 × 10^2^	
	Combination	13	4.07 × 10^2^	4.62 × 10^1^	

*Staphylococcus epidermidis*	Lacalut cleaning tablet	13	6.38 × 10^2^	3.55 × 10^2^	0.795
	Brushing with CHX toothpaste	13	3.23 × 10^2^	6.39 × 10^1^	
	Combination	13	2.80 × 10^2^	6.68 × 10^1^	

Streptococci	Lacalut cleaning tablet	13	1.40 × 10^5^	2.49 × 10^4^	0.496
	Brushing with CHX toothpaste	13	1.34 × 10^5^	3.79 × 10^4^	
	Combination	13	1.02 × 10^5^	1.71 × 10^4^	

Total anaerobic bacteria	Lacalut cleaning tablet	13	7.35 × 10^5^	1.26 × 10^5^	0.405
	Brushing with CHX toothpaste	13	5.64 × 10^5^	7.90 × 10^4^	
	Combination	13	5.16 × 10^5^	5.87 × 10^4^	

**Table 3:** Comparisons of bacterial biofilm on polished and unpolished acrylic tiles of three cleaning methods using the independent *t*-test.

Bacterial strain	Cleaning method	Independent *t*-test			
		Mean difference	df	*T*	Sig. (2-tailed)
Staphylococci	Lacalut cleaning tablet	−0.21	24	−1.580	0.127
	Brushing with CHX toothpaste	−0.85	13.101	−2.474	**0.028**
	Combination	−0.21	19.541	−2.262	**0.035**

*Staphylococcus aureus*	Lacalut cleaning tablet	−0.43	24	−1.206	0.239
	Brushing with CHX toothpaste	−0.10	20.454	−2.479	**0.022**
	Combination	−0.74	12.647	−2.477	**0.028**

*Staphylococcus epidermidis*	Lacalut cleaning tablet	−0.16	24	−0.341	0.736
	Brushing with CHX toothpaste	−0.69	18.760	−1.624	0.121
	Combination	−0.15	22.366	−0.464	0.647

Streptococci	Lacalut cleaning tablet	−0.30	24	−3.287	**0.003**
	Brushing with CHX toothpaste	−0.47	24	−2.872	**0.025**
	Combination	−1.37	12.865	−2.390	**0.033**

Total anaerobic bacteria	Lacalut cleaning tablet	−0.22	24	−1.843	0.078
	Brushing with CHX toothpaste	−0.19	24	−1.205	0.240
	Combination	−0.22	24	−2.565	**0.017**

*P* value <0.05 implies statistically significant difference.

## Data Availability

The data used to support the findings of this study are available from the corresponding author upon request.
